# Systematic review: probiotics for functional constipation in children

**DOI:** 10.1007/s00431-017-2972-2

**Published:** 2017-08-01

**Authors:** Katarzyna Wojtyniak, Hania Szajewska

**Affiliations:** Department of Paediatrics, The Medical University of Warsaw, Żwirki i Wigury 63A, 02-091 Warsaw, Poland

**Keywords:** Probiotics, RCT, Microbiota, Functional gastrointestinal disorders

## Abstract

We updated our 2010 systematic review on the efficacy of probiotics in the treatment of constipation in children. The MEDLINE, EMBASE, and Cochrane Library databases; clinical trial registries; and reference lists of included studies were searched to February 2017 for randomized controlled trials (RCTs) performed in children, with no language restriction. The primary outcome measure was treatment success, as defined by the investigators. We included seven RCTs with a total of 515 participants. Included trials were heterogeneous with respect to study population, probiotic strains, dosages, study duration, and follow-up. Pooled results of two RCTs showed no significant difference between the *Lactobacillus rhamnosus casei* Lcr35 and placebo groups with respect to treatment success. Other probiotics were studied in single trials only. There was no significant difference between the probiotic and control groups with respect to treatment success. While some probiotic strains showed some effects on defecation frequency, none of the probiotics had beneficial effects on frequency of fecal incontinence or frequency of abdominal pain. Adverse events were rare and not serious.

*Conclusion*: Limited evidence does not support the use of any of currently evaluated probiotics in the treatment of functional constipation in children.

**Table:** 

**What is Known:** • *Conventional treatment for functional constipation in children does not always provide satisfying improvement.* • *Probiotics have been suggested as potential treatment modalities for this condition.*
**What is New:** • *Probiotics are ineffective for the management of functional constipation in children in terms of treatment success, frequency of fecal incontinence, and frequency of abdominal pain.*

**Electronic supplementary material:**

The online version of this article (doi:10.1007/s00431-017-2972-2) contains supplementary material, which is available to authorized users.

## Introduction

Functional constipation is common in children with a prevalence ranging between 0.7 to 29.6%, depending on the criteria used [20]. The diagnosis of functional constipation is based on the Rome criteria for functional gastrointestinal disorders, currently the Rome IV criteria [4, 15]. According to the 2014 guidelines developed by the European and North American Societies for Paediatric Gastroenterology, Hepatology and Nutrition (ESPGHAN/NASPGHAN) [27], polyethylene glycol (PEG) is the first-line treatment for children presenting with fecal impaction, and it is also used as maintenance therapy. If PEG is not sufficient, other laxatives may be considered as a second-choice treatment. If PEG is not available, lactulose is recommended. For many patients, however, current treatment options do not provide sustained relief of symptoms. Data have shown that 10% of children with functional constipation take laxatives for longer than 12 months, and 40% are still symptomatic despite use of laxatives [10]. Approximately 50% of children with functional constipation have had at least one relapse within the first 5 years after initial recovery [29]. Therefore, other therapeutic possibilities are being sought.

In adults, experimental studies have shown that constipation is often associated with gut microbiota dysbiosis, consisting of the modified abundance of certain taxa of the colonic microbiome [1]. For example, some data have suggested the decreased abundance of *Bifidobacteria*, *Lactobacillus*, *Bacteroides*, and *Prevotella* [17, 31, 32]. In children, one recent study showed that in those with functional constipation, the most discriminative species were *Bacteroides fragilis*, *Bacteroides ovatus*, *Bifidobacterium longum*, *Parabacteroides* species (increased), and *Alistipes finegoldii* (decreased) [8]. However, it remains to be determined if these alterations are a cause or a consequence of altered gut motility. Considering the potential role of the microbiota, there is a question as to whether modulating the gastrointestinal microbiota plays a role in the management of functional constipation.

Probiotics are defined as live microorganisms that, when administered in adequate amounts, confer a health benefit on the host [14]. There are several mechanisms of action by which probiotics may offer some benefit in the management of functional constipation. First, they modify the altered intestinal microbiota. Second, probiotic metabolites may alter gut sensation and motility function. Finally, some probiotics may regulate the intraluminal environment by increasing the end products of bacterial fermentation, affecting secretion and absorption of water and electrolytes, producing lactate and short-chain fatty acids, and reducing intraluminal pH [31, 32]. In 2010, our team reviewed data on the effectiveness of probiotic supplementation for the treatment of constipation in pediatric or adult populations [6]. We concluded that until more data are available, the use of probiotics for the treatment of constipation should be considered investigational. As new pediatric data have become available, here we report an updated systematic review and meta-analysis on the efficacy and safety of using probiotics for the management of functional constipation in children. Considering that probiotics have strain-specific effects, the focus was on individual probiotic strains, not on probiotics in general.

## Methods

This systematic review was carried out in line with guidelines from the Cochrane Handbook for Systematic Reviews of Interventions [13] and the PRISMA statement for reporting [19]; however, the protocol for this review has not been registered. The lack of registration was because the protocol for our updated systematic review was the same as the one used in our primary review [6].

### Criteria for considering studies for this review

We included randomized controlled trials (RCTs) examining the effects of probiotics compared with placebo, no treatment, or any pharmacological therapy in patients aged 0–18 years with functional constipation diagnosed according to either the authors’ definition or specific diagnostic criteria such as the Rome II, III, or IV criteria [4, 15, 16, 21, 28]. Trials including patients with an organic cause for constipation or with a history of colorectal surgery were excluded. All probiotic strains, doses, treatment regimens, and durations of therapy were considered. Probiotics were administered in capsule, powder, tablet, or fortified food forms. The primary outcome measure was treatment success, as defined by the investigators. The secondary outcome measures were defecation frequency, frequency of fecal incontinence, frequency of abdominal pain (all at the end of the intervention period), and adverse events. Other outcome measures reported by the investigators were also considered, if relevant to the current review.

### Search methods for identification of studies

The MEDLINE (via PubMed (National Library of Medicine), EMBASE, and Cochrane Library databases were searched from May 2009 (end date of last search) to February 2017, with no language restriction. The search terms were as follows: *constipation* AND probiotic*, *Lactobacillus*, *L. GG*, *LGG*, *L. acidophilus*, *L. rhamnosus*, *L. plantarum*, *L. casei*, *L. gasseri*, *L. reuteri*, *L. lactis*, *Bifidobacterium*, *B. breve*, *B. longum*, *B. infantis*, *B. adolescentis*, *B. lactis*, *Bacillus*, *Clostridium butyricum*, *Streptococcus thermophilus*, *Escherichia coli*, *Propionibacterium freundendsreichii*, *Enterococcus SF68*, *Enterococcus faecalis*, *Saccharomyces boulardi*, and *VSL#3*. The search strategy used both keywords and MeSH terms. No other limits were applied to any of the searches.

### Searching other resources

Two registries for clinical trials (www.clinicaltrials.gov, www.clinicaltrialsregister.eu) were screened to identify unpublished and ongoing studies. Moreover, the reference lists of all identified studies were checked as potentially sources of adequate trials.

### Data collection and analysis

The authors carried out the search of the databases, and, for potentially relevant studies, full text copies were obtained. After review of the full texts of these articles, those that fulfilled the inclusion criteria were selected. The authors carried out these stages of the review independently. Disagreements between authors were resolved by discussion to reach a consensus. For the included studies, the authors independently extracted data concerning the methods, settings, participants (age, sex), definitions of constipation, interventions (probiotic strain(s) and species, durations of intervention, doses), comparator groups, outcomes, and results, with the use of standard extraction tables. Disagreements were discussed by the authors in order to reach a consensus.

### Assessment of risk of bias in included studies

The Cochrane Collaboration’s tool for assessing risk of bias was used to establish the risk of bias [24]. In this evaluation, we checked generation of random sequences (selection bias), concealment of allocation (selection bias), blinding of participants and personnel (performance bias), blinding of outcome assessment (detection bias), incomplete outcome data (attrition bias), and selective reporting (reporting bias). Assessment of studies’ methods according to these criteria allowed us to judge risk of bias as high, low, or unclear. Depending on whether each study’s methods fulfilled or did not fulfill these criteria, the risk of bias was judged as low or high, respectively. If information about these factors did not appear in the publication, the risk of bias was determined to be unclear.

### Statistical analysis

For dichotomous outcomes, the total number of patients and the number of patients who experienced the event were extracted, and the risk ratio (RR) and 95% confidence interval (CI) were calculated. For continuous outcomes, the total number of patients was extracted, and the mean differences (MD) with 95% CI were calculated. Each probiotic strain was evaluated separately. *χ* ^2^ and *I* ^2^ were determined to quantify heterogeneity. For *χ* ^2^, a *P* < 0.10 indicated statistical significance for heterogeneity. For *I* ^2^, a rough guide to interpretation is as follows: 0 to 40%: might not be important; 30 to 60%: may represent moderate heterogeneity; 50 to 90%: may represent substantial heterogeneity; 75 to 100%: considerable heterogeneity [13]. All analyses were based on the random effects model. The data were analyzed using Review Manager (RevMan) ([Computer program]. Version 5.3. Copenhagen: The Nordic Cochrane Centre, The Cochrane Collaboration, 2014).

## Results

### Results of the search

For a flow diagram documenting the identification process for the eligible trials, see Fig. S1. Characteristics of the seven included RCTs [2, 5, 7, 12, 22, 25, 30] involving 515 participants (263 in the probiotic group and 252 in the control group) are summarized in Table S1. Compared with our 2010 systematic review, five new publications published subsequently were included. Characteristics of the excluded studies [3, 23, 26] are summarized in Table S2. In addition, four registered trials were identified. All of them have unknown status; the completion date had passed, and the status had not been verified (ClinicalTrials.gov, Identifier: NCT01629147, NCT01913665, NCT01388712, NCT01587846).

All included trials were double-blind RCTs. The sample sizes ranged from 27 to 159 participants. The age range in the different studies varied between 6 months and 16 years. Only *Lactobacillus casei rhamnosus* Lcr35 was studied in two RCTs. The remaining probiotics were tested in single trials only. These included *Lactobacillus rhamnosus* GG; *Lactobacillus reuteri* DSM 17938; *Bifidobacterium lactis* DN-173 010 [and yogurt starter cultures: *Lactobacillus delbrueckii* ssp*. bulgaricus* (CNCM I-1632 and I-1519), *Streptococcus thermophilus* CNCM I-1630, and *Lactococcus cremoris* (CNCM I-1631)]; *B. longum* [and yogurt starters, *Lactobacillus delbrueckii* subspecies *bulgaricus* and *Streptococcus thermophilus* from the YF-L812 commercial culture]; and a mixture of seven strains (*Lactobacillus casei* PXN 37, *Lactobacillus rhamnosus* PXN 54, *Streptococcus thermophilus* PXN 66, *Bifidobacterium breve* PXN 25, *Lactobacillus acidophilus* PXN 35, *Bifidobacterium infantis* PXN 27, and *Lactobacillus bulgaricus* PXN 39). Two studies assessed the effectiveness of using probiotics as an additional therapy to lactulose [2, 22]. The doses of the probiotics used ranged from 1 × 10^8^ to 8.4 × 10^9^ colony-forming units (CFU)/day. The probiotics were provided in oil suspension, capsules, yogurt, or sachets.

**Fig. 1:**
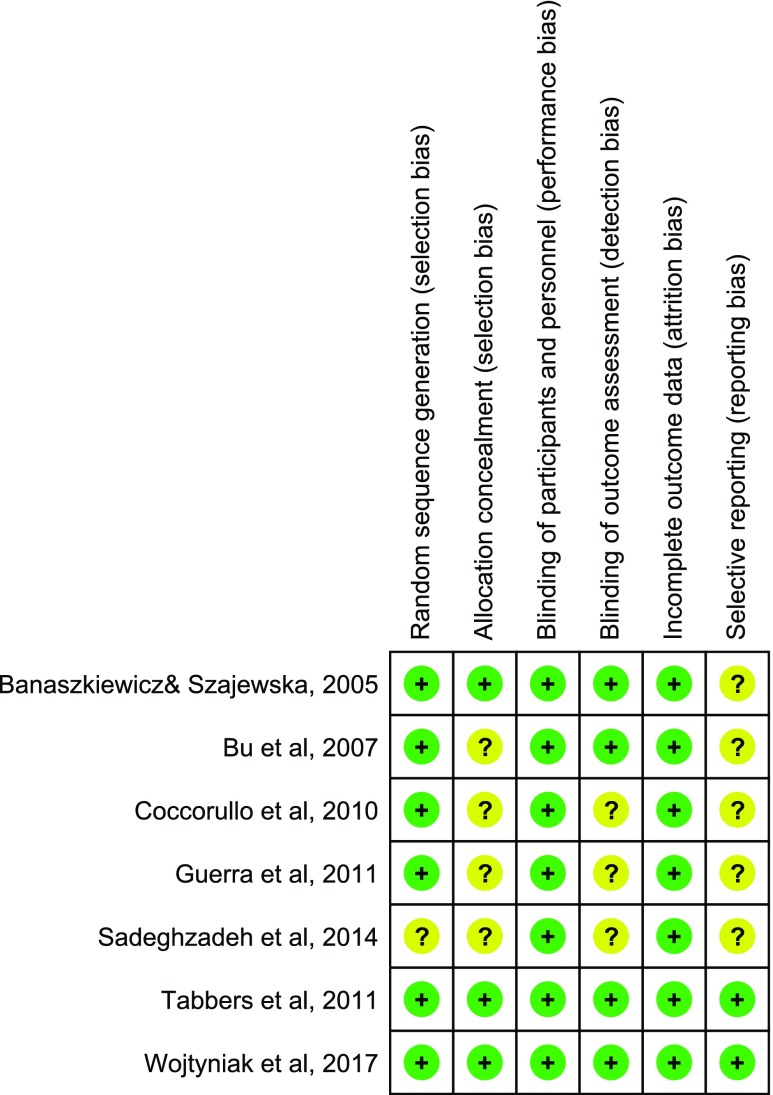
Assessment of methodological quality and potential risk of bias

For the assessment of methodological quality and potential risk of bias, see Fig. 1. Two of the seven included trials were considered of “low risk of bias”. The methodological limitations were unclear random sequence generation (one RCT), unclear allocation concealment (four RCTs), unclear blinding of outcome assessment (three RCTs), and unclear bias due to selective reporting (five RCTs).

### Effects of interventions

A summary of the outcomes is presented in Table 1.

**Table 1:** Summary of the results on the effectiveness of probiotics vs. control

Outcome	Probiotic(s)	Trial(s)	Effect size (95% CI)
Treatment success	*L. casei rhamnosus* Lcr35	2 RCTs (*n* = 108) (Fig. 2)	RR 2.08 (0.19 to 23.37)
	*L.* GG	1 RCT (*n* = 84)	RR 1.06 (0.8 to 1.4)
	*B. lactis* DN 173 010	1 RCT (*n* = 159)	RR 1.14 (0.91 to 1.43)
Defecation frequency	*L. casei rhamnosus* Lcr35	2 RCTs (*n* = 108) (Fig. 3)	MD 0.16 (−4.38 to 4.69)
	*L.* GG	1 RCT (*n* = 84)	MD −0.7 (−1.79 to 0.39)
	*L. reuteri* DSM 17938	1 RCT (*n* = 44)	*P* = 0.027 (data not given)
	*B. lactis* DN-173 010	1 RCT (*n* = 159)	4.5 vs. 3.9; *P* = 0.51 (no data were given)
	*B. longum*	1 RCT (*n* = 59)	*P* = 0.012 (data not given)
	Mixture of 7 probiotics	1 RCT (*n* = 48)	MD 0.54 (0.07 to 1.01)
Frequency of fecal incontinence	*L. casei rhamnosus* Lcr35	2 RCTs (*n* = 108) (Fig. 3)	MD −0.05 (−0.63 to 0.53)
	*L.* GG	1 RCT (*n* = 84)	MD 0.5 (−0.1 to 1.1)
	*B. lactis* DN-173 010	1 RCT (*n* = 159)	36.6% vs. 48.6%, *P* = 0.19
	Mixture of 7 probiotics	1 RCT (*n* = 48, but only subset of children was evaluated)	*P* = 0.125
Frequency of abdominal pain	*L. casei rhamnosus* Lcr35	2 RCTs (*n* = 108) (Fig. 3)	MD −2.13 (−7.12 to 2.87)
	*B. longum*	1 RCT (*n* = 59)	*P* = 0.015 (data not given)
	*B. lactis* DN-173 010	1 RCT (*n* = 159)	58.3% vs. 54.2%, *P* = 0.92
	Mixture of 7 probiotics	1 RCT (*n* = 48, but only subset of children was evaluated)	*P* = 0.161

*MD* mean difference, *RCT* randomized controlled trial, *RR* risk ratio

#### Treatment success (Fig. 2)

Four of the included studies [2, 5, 25, 30] reported on treatment success, defined as defecation at least three times per week and no fecal incontinence. Based on the pooled results of two RCTs (*n* = 108) [5, 30], treatment success was similar in the *L. casei rhamnosus* Lcr35 group and the placebo group (RR 2.08, 95% CI 0.19 to 23.37). Significant statistical heterogeneity was found (*χ* ^2^ = 6.66; *P* = 0.01; *I* ^2^ = 85%). One trial evaluated the effectiveness of *L. rhamnosus* GG [2], and one, *Bifidobacterium lactis* DN-173010 [25]; neither probiotic was statistically significantly more efficacious than placebo.

**Fig. 2:**
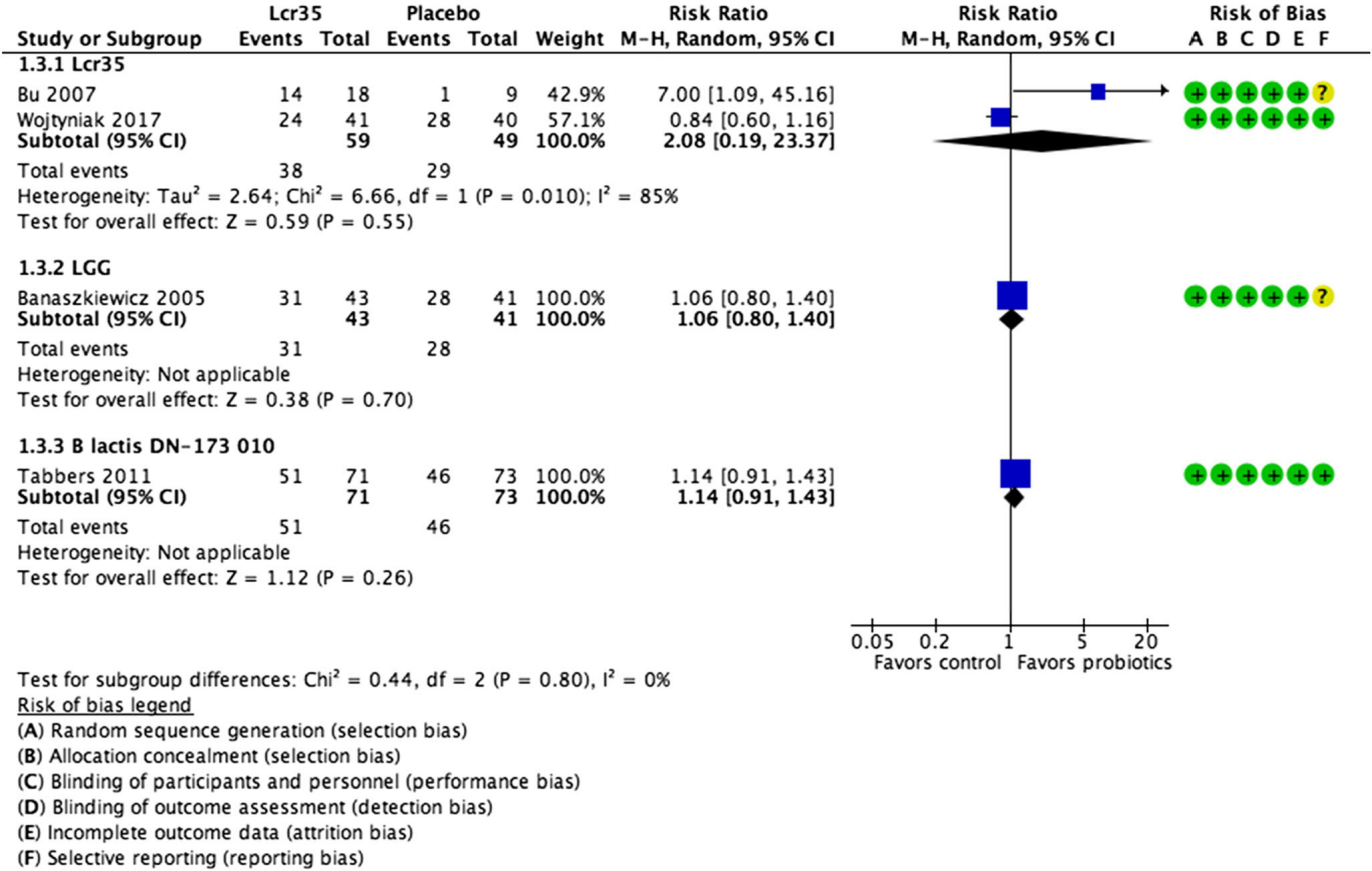
Individual probiotics vs. control for functional constipation in children. Treatment success

#### Defecation frequency

**Fig. 3:**
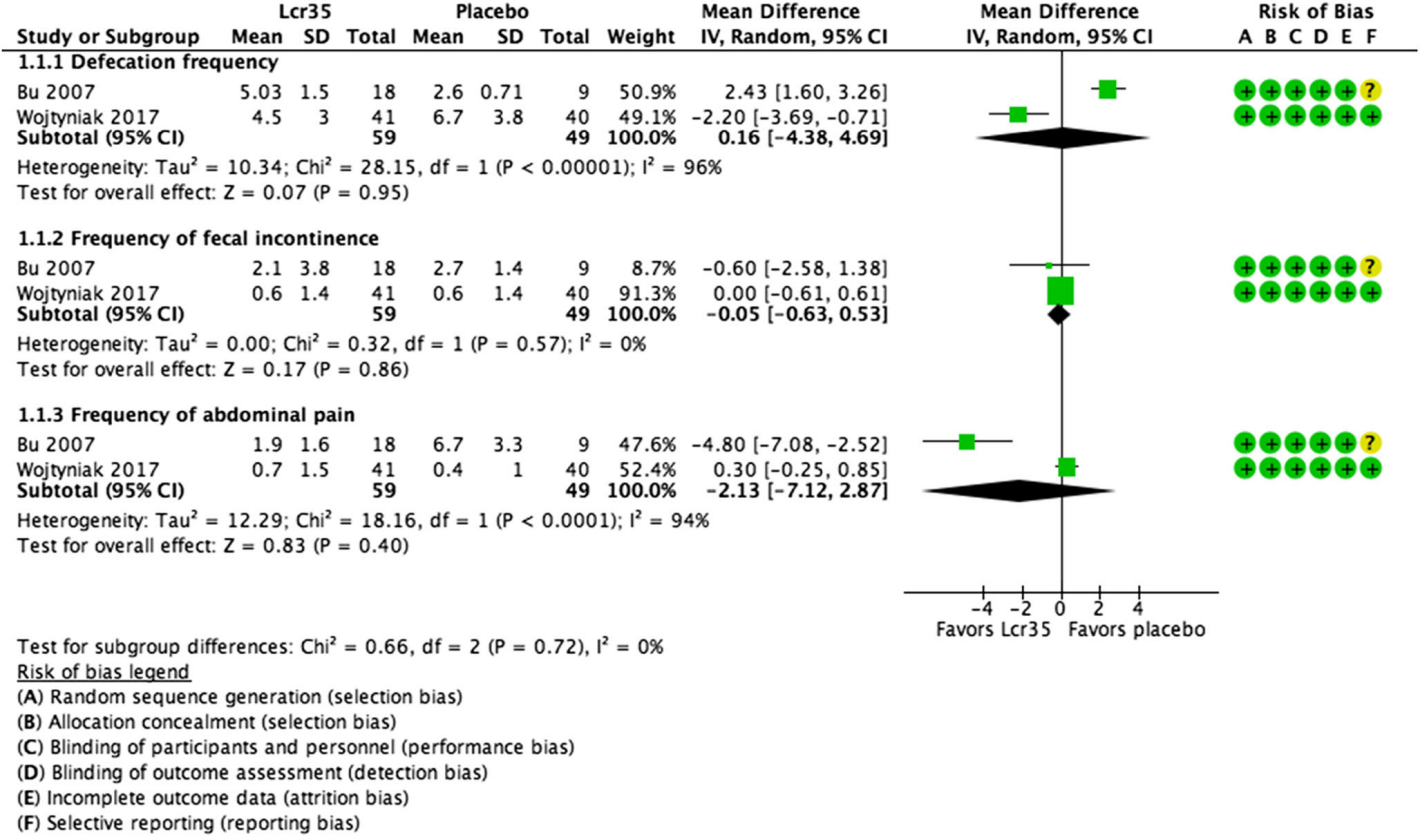
*L. casei rhamnosus* Lcr35 vs. placebo for functional constipation in children. Defecation frequency; frequency of fecal incontinence; frequency of abdominal pain

Stool frequency was measured in all seven studies [2, 5, 7, 12, 22, 25, 30]. Pooled results of two RCTs (*n* = 108) [5, 30] showed no significant difference in defecation frequency in patients treated with *L. casei rhamnosus* Lcr35 compared with those treated with placebo at the last week of the intervention (MD 0.16 defecations per week, 95% CI −4.38 to 4.69); however, significant heterogeneity was found (*χ* ^2^ = 28.15; *P* < 0.00001; *I* ^2^ = 96%) (Fig. 3).

Data from one RCT [7] found that compared with placebo, administration of *L. reuteri* DSM 17938 significantly increased the frequency of bowel movements at the end of the intervention (week 8; *P* = 0.027; no data given in the referenced article).

One RCT [12] showed that compared with placebo, administration of *Bifidobacterium longum* significantly increased the frequency of bowel movements at the end of the intervention (week 10; *P* = 0.012; no data given in the referenced article).

One RCT [2] reported that compared with placebo, administration of *Lactobacillus* GG had no effect on stool frequency in children treated with lactulose (1 RCT, *n* = 84, MD −0.73 bowel movements per week, 95% CI −1.79 to 0.39).

One RCT [25] found that compared with the administration of a control product, the administration of a fermented dairy product containing *Bifidobacterium lactis* DN-173 010 had no effect on stool frequency at the end of intervention (3.9 vs. 4.5 stools per week at week 3, respectively; *P* = 0.51).

One RCT [22] showed that compared with placebo, administration of a mixture of seven probiotic strains had an effect on stool frequency at the end of the intervention (week 4); however, the difference between groups was of borderline statistical significance (1 RCT, *n* = 48, MD 0.54 defecations per week, 95% CI 0.07 to 1.01).

#### Frequency of fecal incontinence

Fecal incontinence was reported in five RCTs [2, 5, 22, 25, 30]. Based on the pooled results of two RCTs (*n* = 108) [5, 30], there was no significant effect of *L. casei rhamnosus* Lcr35 compared with placebo on the frequency of fecal incontinence at the end of intervention (MD −0.05 episodes per week, 95% CI −0.63 to 0.53); no significant heterogeneity was found (*χ* ^2^ = 0.32; *P* = 0.57; *I* ^2^ = 0%) (Fig. 3). No other trials reported significant differences between the probiotics and the placebo groups in the frequency of fecal incontinence. Of note, in the study by Sadeghzadeh et al., fecal incontinence was assessed only in patients who had these symptoms before the intervention. In this subgroup, no significant difference between the control and probiotic groups in the frequency of fecal incontinence was found at the end of the intervention (*P* = 0.125) [22] (Table 1).

#### Frequency of abdominal pain

Abdominal pain was assessed in five RCTs [5, 12, 22, 25, 30]. Pooled results of two RCTs (*n* = 108) [5, 30] showed no difference between the *L. casei rhamnosus* Lcr35 and control groups in the frequency of abdominal pain (MD −2.13, 95% CI −7.12 to 2.87), but the heterogeneity was considerable (*χ* ^2^ = 18.16; *P* < 0.0001; *I* ^2^ = 94%) (Fig. 3).

Among other RCTs, one reported data on use of *Bifidobacterium longum* [12]. There was a significant difference between the control and probiotic groups in the frequency of abdominal pain at the end of the intervention (week 10; *P* = 0.015; no data given in the referenced article).

One RCT reported no significant difference between the control and *Bifidobacterium lactis* DN-173 010 groups in the frequency of abdominal pain at week 3 of the intervention (54.2 vs. 58.3%, respectively, OR 0.97, 95% CI 0.56 to 1.69, *P* = 0.92) [25].

In the study by Sadeghzadaeh et al., abdominal pain was assessed only in patients who had these symptoms before the intervention. In this subgroup, no significant difference between the control and probiotic groups in the frequency of abdominal pain was found at the end of the intervention (*P* = 0.161) [22].

#### Adverse events

Of the seven trials included in the review, six reported on adverse events [2, 5, 7, 22, 25, 30]. In these trials, the probiotics were well tolerated. Adverse events were similar in the experimental and control groups (RR 0.58, 95% CI 0.25 to 1.31). No significant heterogeneity was found (*χ* ^2^ = 1.01; *P* = 0.6; *I* ^2^ = 0%). The most frequently occurring adverse events were abdominal pain, vomiting, and gastroenteritis.

## Discussion

### Summary of evidence

This systematic review demonstrates that probiotics are ineffective for the management of functional constipation in children in terms of treatment success, defecation frequency, frequency of fecal incontinence, and frequency of abdominal pain. Adverse events were rare and not serious.

### Strengths and limitations

One characteristic that makes our meta-analysis distinct from other reviews is that it does not focus on probiotics in general, but rather on individual probiotic strains. We based our systematic review on the methodology developed by the Cochrane Collaboration [13], and we reported data according to the PRISMA statement [19]. The comprehensive literature search, which included searching for not yet published trials, with no restriction by language, reduced the risk that relevant studies were missed. The risk of bias in the included trials was also assessed. However, we are aware of some limitations. While the analyses were defined a priori, the protocol of the review has not been registered. As stated earlier, the lack of registration was because the protocol for our updated systematic review was the same as the one used in our primary review [6]. We included seven RCTs with five different probiotic strains and one mixture of seven probiotic strains. Only *L. casei rhamnosus* Lcr35 was used in two trials, which demonstrated contradictory results with regard to treatment success and defecation frequency.

The doses of the probiotics varied, and probiotics were used as an additional therapy to lactulose in two trials. While most of the studies defined functional constipation according to the Rome III criteria, the investigators in two trials developed their own definition, which can influence the study population. Additionally, there were substantial discrepancies between ages of included children, ranging from infants to adolescents.

An additional limitation of our review is the heterogeneity in outcome measures used in the included trials. The primary outcome measure in our review was treatment success, as defined by the investigators. However, these definitions varied between studies. None of the outcome measures were assessed in all of the included studies in a form that was suitable for a meta-analysis, making comparison difficult. Taken together, our findings must be interpreted with caution.

### Agreement and disagreement with other studies or reviews

The previous review published by our team included only two trials from the pediatric population. This review found that administration of *L. rhamnosus* GG was not effective, while the administration of *L. casei rhamnosus* Lcr35 increased the number of stools and reduced the number of hard stools. The authors emphasized that this conclusion was based on a single study, which had a very small number of participants. The current review included more trials involving more patients and focused on children only. We decided not to include the adult population, because of two recently published, well-designed reviews that evaluated the effectiveness of probiotic administration in adults with functional constipation [9, 11]. A systematic review and meta-analysis by Dimidi et al. [9] involved 14 RCTs (1182 participants). Overall, probiotics significantly reduced whole gut transit time by 12.4 h (95% CI −22.3 to −2.5 h) and increased stool frequency by 1.3 bowel movements/week (95% CI 0.7 to 1.9 bowel movements/week). There was a visible strain-specific effect. In adults, the administration of *B. lactis* increased stool frequency (weighted mean difference, WMD, 1.5 bowel movements/week, 95% CI 0.7 to 2.3 bowel movements/week) and improved stool consistency (standardized mean difference, SMD, 0.46, 95% CI 0.08 to 0.85). These effects were not demonstrated with *L. casei* Shirota. Ford et al. [11] performed a systematic review and meta-analysis to examine the efficacy of treatment with prebiotics, probiotics, and synbiotics in adults with irritable bowel syndrome and chronic constipation. The authors included three RCTs involving 245 patients with chronic constipation. There was no statistically significant effect of probiotics in terms of failure to respond to therapy (RR 0.29, 95% CI 0.07 to 1.12), but probiotics significantly increased the mean number of stools per week (1.49, 95% CI 1.02 to 1.96).

Our findings are in line with the results of a published 2013 review and meta-analysis evaluating the effectiveness of probiotics for the management of childhood functional gastrointestinal disorders [18]. The authors included three RCTs. Probiotics did not have a significant effect with respect to treatment success (RR 1.16, 95% CI 0.83 to 1.62) and defecation frequency (SMD 0.44, 95% CI −0.35 to 1.24).

The lack of an effect of currently studied probiotics does not preclude the possibility that other strains (single or in combinations) will be effective. Both a better understanding of the microbiota differences in constipated and non-constipated children and criteria for the in vitro selection of probiotic microorganisms for further clinical trials are needed. Statistically well-powered RCTs should have relevant inclusion/exclusion criteria, validated clinical outcome measures (with definitions), and effect sizes reported in a clinically meaningful way.

## Conclusion

Current limited evidence does not support the use of probiotics in the treatment of functional constipation in children. The findings of this meta-analysis support current ESPGHAN/NASPGHAN recommendations that probiotics should not be used in the treatment of functional constipation in children [27].

## Electronic supplementary material

Fig S1 (DOCX 36 kb)

Table S1 (DOCX 19 kb)

Table S2 (DOCX 15 kb)

## Abbreviations

*CFU*: Colony-forming units
*CI*: Confidence interval
*MD*: Mean difference
*ESPGHAN*: European Society for Paediatric Gastroenterology, Hepatology and Nutrition
*NASPGHAN*: North American Society for Pediatric Gastroenterology, Hepatology and Nutrition
*PEG*: Polyethylene glycol
*PRISMA*: Preferred Reporting Items for Systematic Review and Meta-Analyses
*RCT*: Randomized controlled trial
*RR*: Risk ratio
*SMD*: Standardized mean difference
