# Supplementary figures and images for: Systematic review: probiotics for functional constipation in children
Source: Eur J Pediatr. 2017 Aug 1;176(9):1155–62. doi: 10.1007/s00431-017-2972-2 (PMC5563334; doi:10.1007/s00431-017-2972-2)

**Fig. S1** Flow chart of included trials.


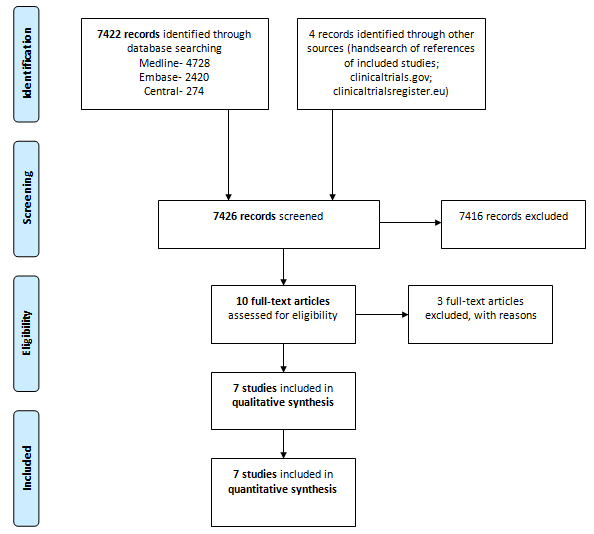

Supplement: Supplementary file 1 — (DOCX 36 kb) [file 431_2017_2972_MOESM1_ESM.docx]
