# Supplementary material for: Systematic review: probiotics for functional constipation in children
Source: Eur J Pediatr. 2017 Aug 1;176(9):1155–62. doi: 10.1007/s00431-017-2972-2 (PMC5563334; doi:10.1007/s00431-017-2972-2)
Supplement: Supplementary file 2 — (DOCX 19 kb) [file 431_2017_2972_MOESM2_ESM.docx]

**Table S1.** Characteristics of the included studies.

| Study (location) | Intervention | Comparison | Exp/cont  (Age) | Definition of constipation | Duration of intervention | Outcomes | Funding | Product  manufacturer |
| --- | --- | --- | --- | --- | --- | --- | --- | --- |
| Banaszkiewicz & Szajewska, 2005 (Poland) [2] | *L. rhamnosus* GG  (2x10^9^ CFU)  + lactulose | Placebo  + lactulose | 43/41  (2-16 y) | <3 BMs per week during for at least 12 weeks | 12 wk | Treatment success (≥3 spontaneous BMs per week with no episodes of fecal soiling)  Number of BMs per week  Number of episodes of fecal soiling per week  Straining frequency per week  Stool consistency | No information given | Dicofarm, Rome, Italy |
| Bu et al, 2007 (Taiwan) [5] | *L. casei rhamnosus* Lcr35 (8x10^8^ CFU) | Placebo | 18/9 (<10 y) | <3 BMs per week for >2 months plus at least one of the criteria: anal fissures with bleeding due to constipation, fecal soiling, or passage of large and hard stool. | 4 wk | Treatment success (≥3 spontaneous BMs per week with no episodes of fecal soiling in the fourth week)  Number of BMs per week  Number of episodes of fecal soiling per week  Number of episodes of abdominal pain per week  Stool consistency  Use of lactulose or enema | No information given | Laboratoires Lyocentre, Aurillac, France |
| Coccorullo et al, 2010 (Italy) [7] | *L. reuteri* DSM 17938 (1x10^8^ CFU) | Placebo | 22/22 (>6 mo) | Rome III criteria | 8 wk | Frequency of BMs per week (results presented in a graph, actual number not reported)  Stool consistency (results presented in a graph, actual number not reported)  Presence of inconsolable crying episodes (results presented in a graph, actual number not reported) | Noos, Italy | Reuterin, Noos S.r.l.;  BioGaia AB, Stockholm, Sweden |
| Guerra et al, 2011 (Brazil) [12] | A goat yogurt (1 ml) containing *B. longum* 10^9^CFU, in addition to yogurt starters (*L delbrueckii subspecies bulgaricus* and *Streptococcus thermophilus* from the YF-L812 commercial culture] | Goat yogurt | 29/30  (5-15 y) | Rome III criteria | 10 wk (crossover after 5wk) | Change in defecation frequency (results presented in a graph, actual number not reported)  Stool consistency (results presented in a graph, actual number not reported)  Abdominal pain (results presented in a graph, actual number not reported)  Defecation pain (results presented in a graph, actual number not reported) | Grants from Conselho Nacional de Desenvolvimento Cientifico e Tecnológico and Fundação de Amparo à Pesquisa do Estado de Minas Gerais | Difco, Sparks, United States |
| Sadeghzadeh et al, 2014 (Iran) [22] | *L. casei* PXN 37, *L. rhamnosus* PXN 54, *Str. thermophilus* PXN 66, *B. breve* PXN 25, *L. acidophilus* PXN 35, *B. infantis* PXN 27, *L. bulgaricus* PXN 39)  (1x10^9^ CFU)  + lactulose | Placebo  + lactulose | 24/24  (4-12 y) | Rome III criteria | 4 wk | Frequency of defecation  Stools consistency  Frequency of fecal incontinence episodes  Frequency of abdominal pain  Adverse effects  Weight gain | Research Department of Zanjan University of Medical Sciences | Nikooteb Company, Tehran, Iran |
| Tabbers et al, 2011 (Netherlands & Poland) [25] | A fermented dairy product containing *B. lactis* DN- 173 010 [and yogurt starter cultures: *Lactobacillus delbrueckii* ssp*. bulgaricus* (CNCM I-1632 and I-1519)] (Yogurt, 125-g pot, at least 4.25x10^9^ CFU per pot, 2 pots a day) | Control product (milk-based, non-fermented dairy product), 125-g pot | 79/80  (3-16 y) | Rome III criteria | 3 wk | Change in stool frequency  Rate of success (≥3 BMs per week and <1 fecal incontinence episodes in 2 weeks over the last 2 weeks of product consumption)  Rate of responders (responder is a subject who reports a stool frequency ≥3 episodes during the last week of product consumption)  Frequency of defecation  Stools consistency  Frequency of fecal incontinence episodes  Frequency of pain during defecation  Frequency of digestive symptoms (abdominal pain, flatulence)  Frequency of adverse effects (nausea, diarrhea, bad taste)  Frequency of use of Bisacodyl | Danone Research | Danone, Paris, France |
| Wojtyniak et al, 2017 (Poland) [30] | *L. casei rhamnosus* Lcr35, 8x10^8^ CFU, twice daily | Placebo | 48/46 (<5 y) | Rome III criteria | 4 wk | Treatment success (≥3 spontaneous BMs per week with no episodes of fecal soiling in the fourth week)  Stool consistency  Frequency of defecation  Frequency of fecal soiling  Frequency of pain during defecation  Frequency of abdominal pain or flatulence  Need for intake of additional laxative treatment  Adverse events | Medical University of Warsaw; study product by Sequoia, Warsaw, Poland | Biose, Arpajon sur Cere, France |

exp- experimental, cont- control, y- years, mo- month, wk- week
