# Supplementary material for: Systematic review: probiotics for functional constipation in children
Source: Eur J Pediatr. 2017 Aug 1;176(9):1155–62. doi: 10.1007/s00431-017-2972-2 (PMC5563334; doi:10.1007/s00431-017-2972-2)
Supplement: Supplementary file 3 — (DOCX 15 kb) [file 431_2017_2972_MOESM3_ESM.docx]

**Table S2.** Characteristics of the excluded studies.

| **Study** | **Reason for exclusion** |
| --- | --- |
| Saneian et al. 2013 [23] | Controlled trial. No randomization. |
| Tabbers et al. 2011 [26] | Case series. No randomization. |
| Bekkali et al. 2007 [3] | Case series. No randomization. |
